# Clinical correlates of women endorsing premenstrual suicidal ideation: a cross-sectional study

**DOI:** 10.1186/s13030-022-00252-3

**Published:** 2022-11-08

**Authors:** Sara V. Carlini, Sandra J. Weiss, Lauren Mordukhaev, Sunu Jacob, Heather A. Flynn, Kristina M. Deligiannidis

**Affiliations:** 1 Department of Psychiatry, Maimonides Medical Center, Brooklyn, NY 11219 USA; 2 Department of Community Health Systems, University of California, San Francisco, CA 94143 USA; 3 Donald and Barbara Zucker School of Medicine at Hofstra/Northwell, Hempstead, NY 11549 USA; 4 Division of Psychiatry Research, Department of Psychiatry, Zucker Hillside Hospital, Northwell Health, 75-59 263rd Street, Glen Oaks, NY 11004 USA; 5 Department of Behavioral Sciences and Social Medicine, Florida State University College of Medicine, Tallahassee, FL 32306 USA; 6 Departments of Psychiatry, Molecular Medicine and Obstetrics & Gynecology, Donald and Barbara Zucker School of Medicine at Hofstra/Northwell, Hempstead, NY 11549 USA; 7 Feinstein Institutes for Medical Research, Manhasset, NY 11030 USA; 8 Department of Psychiatry, University of Massachusetts Chan Medical School, Worcester, MA 01655 USA

**Keywords:** Premenstrual dysphoric disorder, Premenstrual syndrome, Suicide, Trauma

## Abstract

**Background:**

Prevalence of premenstrual syndrome (PMS) may be as high as 13-18%, but it remains under-recognized and is associated with increased suicidal ideation (SI), plans, and attempts in epidemiological studies. The present study reports on women endorsing premenstrual SI (PMSI) and characterizes this at-risk group and its clinical correlates.

**Methods:**

A cross-sectional study assessed demographics, anxiety and depression severity, psychiatric diagnoses, menstrual symptoms, SI, and trauma in adult women at a major medical center over 11 months.

**Results:**

Three hundred two women were assessed. Of 153 participants endorsing premenstrual symptoms, 41 (27%) reported new or worsening concurrent premenstrual passive or active SI. Women who reported PMSI were significantly more likely to be single, unemployed, and childless as well as significantly more likely to report interference from premenstrual symptoms, histories of psychiatric hospitalization, adverse childhood events, suicide attempts, and current and past depression and anxiety compared to women without PMSI. The final regression model indicated the most significant predictors of PMSI were history of a depression diagnosis, severity of current depressive symptoms, and having experienced 3 or more childhood adverse events.

**Conclusion:**

Nearly one-third of women reporting premenstrual symptoms endorsed concurrent SI, a clinically valuable demonstration of the importance of this predictable cyclic risk factor.

**Supplementary Information:**

The online version contains supplementary material available at 10.1186/s13030-022-00252-3.

## Background

Premenstrual syndrome (PMS) refers to the physical, cognitive, or emotional changes that arise in a subset of women immediately prior to the onset of menses and that range from mild to severe and disabling [1]. Common symptoms include affective lability (e.g. increased sensitivity to rejection or mood swings), irritability, depressed mood, anxiety, loss of interest in activities, difficulty concentrating, fatigue, hypersomnia or insomnia, changes in appetite (including specific food cravings), and physical symptoms such as muscle pain, breast swelling or tenderness, or bloating [2]. Epidemiological studies have concluded that the prevalence of premenstrual symptoms causing clinically significant interference or distress may be as high as 13-18% [3] and comparable with other depressive disorders [3]. PMS has been significantly associated with an increase in suicidal ideation (SI) but not suicide attempts in a recent meta-analysis [4]. Premenstrual dysphoric disorder (PMDD), a severe form of PMS with strictly defined criteria codified by the American Psychiatric Association in the Diagnostic and Statistical Manual of Mental Disorders Version 5 [2], has additionally been associated with SI, plans to suicide, and suicide attempts in meta-analyses [4, 5].

While the definitive pathophysiology of PMS has not yet been established, several lines of evidence support the theory that neuroactive metabolites of the ovarian steroid hormone progesterone, such as allopregnanolone, which rises steeply in the late luteal phase, exert an effect on GABA-A receptors in the brain to produce negative mood symptoms in susceptible women [6, 7]. Altered sensitivity to the neuroactive steroid allopregnanolone has been found in women with PMDD [8]. Preliminary research has isolated a history of childhood abuse and trauma as one potential antecedent of this altered sensitivity to ovarian steroid hormones seen in women with PMDD [9]. Overall, research suggests that higher trauma burdens, or higher numbers of adverse childhood experiences, may predispose to worse health outcomes, with one study showing that 3 or more adverse events in childhood was associated with worse physical and mental health outcomes in a diverse racial and ethnic sample [10].

Given the relative scarcity of data exploring the relationships between premenstrual symptoms, SI, and history of trauma, the aims of the present study were to assess whether SI was reported to co-occur with any premenstrual symptoms, to characterize the emergent group of women endorsing premenstrual suicidality, and to evaluate for any differences in early trauma and adversity reported by women endorsing premenstrual suicidality and those who did not in an ongoing large, cross-sectional study of mood across the reproductive life cycle. Based on the prior research discussed above, we hypothesized that a discrete group of subjects would endorse SI co-occurring with premenstrual symptoms. We further hypothesized that this group would be more likely to endorse a history of childhood trauma.

## Methods

This study was part of a larger research effort being conducted by the Women’s Mood Disorder Task Group of the National Network of Depression Centers [11]. As part of that research, the Women’s Mood Disorder Task Group collectively developed a questionnaire to clinically characterize women’s mood and related factors across the reproductive life cycle in diverse settings [12]. The self-report questionnaire included questions on demographics, past psychiatric diagnoses, current mood and anxiety symptoms, past stressors, and questions pertaining to mood, impairment, and past treatment in the premenstrual period. The existing questionnaire was a consensus data collection tool across sites, and its existing variables could not be modified. To address our aims, at our data collection site, two questions assessing passive and active SI in the premenstrual period were added to the menstrual mood section in such a way as to not disrupt the existing structure of the core questionnaire. The questions were as follows: “when you are experiencing these symptoms in the week leading up to your period, have you ever also experienced new or increased passive suicidal thoughts (for example: “I wish I were dead,” “I wish I could never wake up,” or “I would be better off dead”)?” and “when you are experiencing these symptoms in the week leading up to your period, have you ever also experienced new or increased active suicidal thoughts (for example: “I want to kill myself,” thinking of plans or means to kill oneself, or thinking of preparatory acts such as writing goodbye notes or making a will)?”

The presence of premenstrual symptoms was assessed by a positive response to a premenstrual symptoms screening question derived from the DSM5 criteria for the diagnosis of PMDD included in the premenstrual mood section of the self-report questionnaire administered to subjects (Table 1). An additional variable was created for women who endorsed premenstrual symptoms with significant interference, which was measured by the question: “Did any of these symptoms make you less productive or efficient at work, school, or home? Interfere with your usual activities, hobbies or social activities? Interfere in your relationships with others?” Current anxiety symptoms were assessed using the validated Generalized Anxiety Disorder 7-item scale (GAD-7) [13], and current depressive symptoms were assessed using the validated Patient Health Questionnaire (PHQ-9) [14]. The Adverse Childhood Experiences (ACEs) Questionnaire was also included to assess childhood experiences of abuse and neglect [15]. A variable was created from the total ACEs score to characterize subjects as high-trauma (ACEs score 3 or greater) and low-trauma (ACEs score 2 or fewer) groups.

**Table 1:** Premenstrual symptoms screening [12]

Have you ever had a year when most of your periods included at least one of the following symptoms in the week leading up to your period?	
- feeling depressed, sad, down, blue, hopeless, worthless or guilty?	
- feeling anxious, tense, or on edge?	
- sudden mood swings or more sensitivity to what others say or do?	
- feeling angry or irritable?	
- feeling less interested in your usual activities?	
- difficulty concentrating?	
- feeling lethargic, tired, fatigued, or lacking in energy?	
- having an increased appetite, overeating, or food cravings?	
- sleeping more than usual, napping, difficulty getting out of bed, or difficulty falling or staying asleep?	
- feeling overwhelmed, unable to cope, or out of control?	
- physical symptoms such as breast tenderness, breast swelling, a bloated sensation, weight gain, headache, joint or muscle pain?	

Table 1 shows the premenstrual symptoms screening question provided to participants as part of the menstrual mood questionnaire

The present analysis encompasses data collected from July 2018 to May 2019 at our academic medical center in metro New York including ambulatory psychiatry and obstetrics-gynecology clinics, inpatient psychiatric units, and the psychiatric partial hospitalization program. Adult women aged 18 and older utilizing these clinical services were approached by research or clinical staff and offered a one-time questionnaire that collected no identifying data. This research protocol was deemed exempt following review by our Institutional Review Board (IRB). A waiver of authorization was provided by the IRB with the relevant US Code of Federal Regulations numbered guideline on which the exemption was based. REDCap data management software was used for data entry and storage [16, 17].

Frequencies were used to characterize the entire sample. A chi-square goodness of fit was used to ascertain whether there were any significant differences between subjects who completed the menstrual mood section of the questionnaire and subjects who did not. A chi-square goodness of fit was also used to test for significant differences in categorical variables in those who reported SI with premenstrual symptoms versus those who did not. Normality of distribution was tested in scale variables using Q-Q plots [18] and the Shapiro-Wilk test [19]. A Mann-Whitney U test [20] was used to test for significant differences in total GAD-7 score and total PHQ-9 score in those who reported SI with premenstrual symptoms versus those who did not. Cohen’s d was calculated for significant results to provide an effect size [21]. All analyses were corrected for multiple comparisons using the Bonferroni correction.

To examine our aim related to trauma, all clinical and demographic variables showing a significant bivariate relationship to reported PMSI were included in a set of logistic regression models until a best-fitting model was identified. This allowed us to examine the relationship of a woman’s history of adverse childhood events to PMSI while adjusting for the effects of other key variables. Collinearity diagnostics were also assessed to determine if any predictors were so highly correlated that their collinearity could reduce precision of the estimated coefficients in the model. SPSS Statistics Version 27 (IBM, Armonk, NY, USA) was used for statistical analysis.

## Results

### Demographics

From July 2018 to May 2019, 302 questionnaires were administered at our academic site. All available data was analyzed. Most women were encountered in outpatient obstetrics-gynecology offices (44%). The majority were aged 26 to 35 (34%), Caucasian (57%), not Hispanic or Latino (72%), married (41%), employed full-time (39%), and had biological children (52%). The largest group of women had a high school diploma (27%) and an income from $101,000 to over $150,000 (43%). The majority denied a history of anxiety (60%) but did report a history of depression (52%), psychiatric hospitalizations (61%), and suicide attempt (75%). Most were menstruating (57%) with regular periods (73%) and with premenstrual symptoms (67%) that interfere (64%) with their functioning (Table 2).

**Table 2:** Participant characteristics

Variables	Frequency (%)
**Age,** ***n*** **= 295**	
18 to 25	67 (23%)
26 to 35	100 (34%)
36 to 45	72 (24%)
46 to 55	23 (8%)
55+	33 (11%)
**Setting,** ***n*** **= 286**	
Inpatient Psychiatry	82 (29%)
Outpatient Psychiatry	78 (27%)
Outpatient Ob-gyn	126 (44%)
**Ethnicity,** ***n*** **= 275**	
Not Hispanic or Latino	199 (72%)
Hispanic or Latino	76 (28%)
**Race,** ***n*** **= 249**	
Caucasian	141 (57%)
African-American	54 (22%)
American Indian	4 (2%)
Asian Indian	20 (8%)
East or South Asian	29 (12%)
Pacific Islander	1 (< 1%)
Other	0 (0%)
**Marital Status,** ***n*** **= 300**	
Single	107 (36%)
Married	123 (41%)
Committed relationship	36 (11%)
Separated	7 (2%)
Widowed	8 (3%)
Divorced	19 (6%)
**Has biological children,** ***n*** **= 299**	
Yes	156 (52%)
No	143 (48%)
**Education,** ***n*** **= 287**	
< High School degree	3 (1%)
High School	77 (27%)
GED	7 (2%)
2-year college (Associate’s degree)	61 (21%)
4-year college (BA, BS)	74 (26%)
Master’s	49 (17%)
Professional (MD, JD)	15 (5%)
PhD	1 (< 1%)
**Income,** ***n*** **= 272**	
< $15,000 - 20,999	57 (21%)
$21,000 - $50,999	35 (13%)
$51,000 - $100,999	63 (23%)
$101, 000 - > $150,000	117 (43%)
**Employment,** ***n*** **= 279**	
Homemaker	33 (12%)
Unemployed	72 (26%)
Employed Occasionally	9 (3%)
Employed Part-time	57 (20%)
Employed Full-time	108 (39%)
**Menstrual status,** ***n*** **= 280**	
Menstruating	159 (57%)
Not menstruating	121 (40%)
**Menstrual regularity,** ***n*** **= 160**	
Regular periods	116 (73%)
Irregular periods	44 (28%)
**Premenstrual symptoms,** ***n*** **= 246**	
Yes	165 (67%)
No	81 (33%)
**Interference from Premenstrual symptoms,** ***n*** **= 157**	
Yes	101 (64%)
No	56 (36%)
**Lifetime history of depression,** ***n*** **= 283**	
Yes	147 (52%)
No	136 (48%)
**Lifetime history of anxiety,** ***n*** **= 272**	
Yes	110 (40%)
No	162 (60%)
**History of suicide attempt,** ***n*** **= 275**	
Yes	70 (26%)
No	205 (75%)
**History of psychiatric hospitalization,** ***n*** **= 275**	
Yes	107 (39%)
No	168 (61%)

### Premenstrual mood

#### Aim 1: premenstrual SI

Two hundred forty-six of the 302 participants provided data for the premenstrual mood section. There were no significant differences between subjects who provided data for the premenstrual mood section and those who did not in the demographic variables listed in Table 2 (Supplementary Table 1). 62% (153/246) answered “yes” to the premenstrual symptom screening question (Table 1). Of those, 26% (41/153) of women reported passive or active SI concurrent with premenstrual symptoms. 21% (31/151) of women specifically reported active suicidal intent concurrent with premenstrual symptoms.

#### Aim 2: correlations with premenstrual SI

##### Demographic variables

After correcting for multiple comparisons, participants reporting concurrent passive or active SI with premenstrual symptoms, or premenstrual SI (PMSI), were significantly more likely to be younger (X^2^ (4, *N* = 153) = 18.465, *p* = 0.02), to be encountered in the inpatient setting (X^2^ (2, *N* = 146) = 33.694, *p* <  0.001), to be single (X^2^ (5, *N* = 153) = 21.119, *p* = 0.01), to not have biological children (X^2^ (1, *N* = 153) = 18.040, *p* <  0.001), to be unemployed or underemployed (X^2^ (4, *N* = 150) = 18.148, p = 0.02), to have been psychiatrically hospitalized in the past (X^2^ (1, *N* = 152) = 37.092, *p* <  0.001), to have a history of suicide attempt (X^2^ (1, *N* = 151) = 26.301, *p* <  0.001), to have a history of depression (X^2^ (1, *N* = 152) = 32.231, *p* <  0.001) and anxiety (X^2^ (1, *N* = 149) = 16.867, *p* <  0.001), and to report significant interference in daily functioning with their menstrual symptoms (X^2^ (1, *N* = 150) = 17.595, *p* <  0.001) compared to participants who denied PMSI (Table 3).

**Table 3:** Demographics of premenstrual symptom group +/− SI

Variables	With SI Frequency (%)	Without SI Frequency (%)	p
**Age,** ***n*** **= 153**			**0.020**^**a**^
18 to 25	19 (51%)	18 (49%)	
26 to 35	9 (15%)	50 (85%)	
36 to 45	9 (23%)	31 (76%)	
46 to 55	4 (36%)	7 (64%)	
55+	0 (0%)	6 (100%)	
**Setting,** ***n*** **= 146**			**0.001**^**a**^
Inpatient Psychiatry	28 (68%)	25 (24%)	
Outpatient Psychiatry	11 (27%)	24 (23%)	
Outpatient Ob-gyn	2 (5%)	56 (53%)	
**Ethnicity,** ***n*** **= 145**			0.711
Not Hispanic or Latino	24 (62%)	83 (78%)	
Hispanic or Latino	15 (39%)	23 (22%)	
**Race,** ***n*** **= 127**			3.664
Caucasian	24 (73%)	55 (59%)	
African-American	8 (24%)	17 (18%)	
American Indian	0 (0%)	3 (3%)	
Asian Indian	0 (0%)	7 (7%)	
East or South Asian	1 (3%)	11 (12%)	
Pacific Islander	0 (0%)	1 (1%)	
**Marital Status,** ***n*** **= 153**			**0.013**^**a**^
Single	25 (61%)	37 (33%)	
Married	4 (10%)	49 (44%)	
Committed relationship	8 (20%)	18 (16%)	
Separated	0 (0%)	4 (4%)	
Widowed	0 (0%)	1 (1%)	
Divorced	4 (10%)	3 (3%)	
**Has biological children,** ***n*** **= 153**			**< 0.001**^**a**^
Yes	9 (22%)	68 (61%)	
No	32 (78%)	44 (39%)	
**Education,** ***n*** **= 152**			0.435
High School	17 (42%)	27 (24%)	
GED	0 (0%)	5 (5%)	
2-year college	13 (32%)	20 (18%)	
4-year college	8 (20%)	33 (30%)	
Master’s	2 (5%)	20 (18%)	
Professional (MD, JD)	1 (2%)	6 (5%)	
**Income,** ***n*** **= 147**			0.108
< $15,000 - 20,999	13 (33%)	18 (17%)	
$21,000 - $50,999	10 (25%)	11 (10%)	
$51,000 - $100,999	6 (15%)	21 (20%)	
$101, 000 - > $150,000	11 (28%)	57 (53%)	
**Employment,** ***n*** **= 150**			**0.020**^**a**^
Homemaker	5 (12%)	10 (9%)	
Unemployed	18 (44%)	18 (17%)	
Employed Occasionally	2 (5%)	4 (4%)	
Employed Part-time	10 (24%)	25 (23%)	
Employed Full-time	6 (15%)	52 (48%)	
**Interference from Premenstrual Symptoms,** ***n*** **= 150**			**< 0.001**^**a**^
Yes	37 (90%)	58 (53%)	
No	4 (10%)	51 (47%)	
**Lifetime history of depression,** ***n*** **= 152**			**< 0.001**^**a**^
Yes	40 (98%)	52 (47%)	
No	1 (2%)	59 (53%)	
**Lifetime history of anxiety,** ***n*** **= 149**			**< 0.001**^**a**^
Yes	31 (76%)	41 (38%)	
No	10 (24%)	67 (62%)	
**History of suicide attempt,** ***n*** **= 151**			**< 0.001**^**a**^
Yes	26 (65%)	23 (21%)	
No	14 (35%)	88 (79%)	
**History of psychiatric hospitalization,** ***n*** **= 152**			**< 0.001**^**a**^
Yes	36 (90%)	38 (34%)	
No	4 (10%)	74 (66%)	
**Trauma History,** ***n*** **= 124**			**0.017**^**a**^
High trauma	25 (49%)	26 (51%)	
Low trauma	9 (12%)	64 (88%)	

^a^Bonferroni adjusted *p* values

Participants who reported PMSI did not differ significantly by income (X^2^ (3, *N* = 147) = 12.329, *p* = 0.11), education X^2^ (5, *N* = 152) = 12.772, *p* = 0.44), ethnicity (X^2^ (1, *N* = 145) = 4.143, *P* = 0.71), or race (X^2^ (5, *N* = 127) = 34.040, *p* = 3.66) (Table 3).

##### Psychiatric symptom scales

Participants who reported PMSI had significantly higher current anxiety on their GAD-7 score (SI mean = 14.8 ± 5.3, No SI mean = 7.2 ± 5.9, U = 5.988, *p* <  0.001, Cohen’s d = − 0.63) and higher current depression based on their PHQ-9 scores (SI mean = 19.1 ± 6.1, No SI mean = 7.2 ± 6.9; U = 7.145, *p* <  0.001, Cohen’s d = − 0.84) than those who did not (Table 4).

**Table 4:** Standardized rating and symptom scale scores in women with premenstrual symptoms +/− SI

	With SI Mean (SD)	Without SI Mean (SD)	p	Cohen’s d
**GAD-7 (*n*** **= 139)**	14.8 (5.3)	7.2 (5.9)	**< 0.001**^**a**^	−0.630
**PHQ-9 (*n*** **= 141)**	19.1 (6.1)	7 (6.9)	**< 0.001**^**a**^	−0.842

*SD* standard deviation

^a^Bonferroni adjusted *p* values

#### Aim 3: differences in early trauma and adversity

Participants reporting PMSI were significantly more likely to be in the high-trauma group, reporting 3 or more adverse events during childhood (X^2^ (1, *N* = 124) = 20.31, *p* = 0.017) (Table 3). 25/51 (51%) of women in the high-trauma group endorsed SI compared to just 9/73 (12%) in the low-trauma group. Based on our findings for the first two aims, thirteen variables showing bivariate relationships to PMSI were included in an integrated logistic regression analysis. Only four of these variables retained their significant relationship with PMSI in the integrated model. These were having a history of a depression diagnosis (B = 4.24, *p* = .01), history of an anxiety diagnosis (B = − 2.99. *p* = .05), current depressive symptoms (B = .21, *p* = .02), and current anxiety symptoms (B = .16, p = .05). All other variables ranged from probability levels of *p* = .13 (employment status) to *p* = .76 (having other biological children). Collinear diagnostics for the covariates did not indicate significant problems with collinearity among the variables. Only two variables showed the potential for a collinear relationship: current depressive symptoms and current anxiety symptoms. However, their Variance Inflation Factors were below the threshold for concern (3.70 and 3.11) [22, 23]. In addition, the condition indices of the eigenvalues in the model were 10 or less.

Based on this initial modeling, the final logistic regression analysis included history of a depression diagnosis, history of an anxiety diagnosis, current anxiety symptoms, current depressive symptoms, and childhood trauma. Table 5 indicates that both a history of anxiety and women’s current anxiety symptoms were ultimately not significant predictors of PMSI. But depression had a significant association with PMSI, including history of a depression diagnosis and current depressive symptoms. Women with a history of depression were approximately 13 times more likely to experience PMSI than other women. Similarly, for current depressive symptoms, the odds that women would experience PMSI increased by 17% for each one-point increase in their severity score on the PHQ-9. After adjusting for these covariates, exposure to childhood trauma did predict PMSI. Women who had 3 or more adverse childhood events were 3.45 times more likely to experience PMSI (Table 5).

**Table 5:** Logistic regression

	B	SE	OR	p	95% CI
**History of Depression Diagnosis**___________	2.57	1.27	13.13	**0.04**	1.090, 15.820
**History of Anxiety Diagnosis**	−.72	.73	.48	0.32	0.116, 2.026
**Childhood Trauma**	1.24	.38	3.45	**0.04**	1.045, 11.414
**Current Anxiety Symptoms**	.03	0.07	1.03	0.69	0.885, 1.202
**Current Depressive Symptoms**	0.15	0.06	1.17	**0.01**	1.028, 1.329

*SE* standard error, *OR* odds ratio, *CI* confidence interval

## Discussion

The aims of the present study were to assess whether women reported SI co-occurring with premenstrual symptoms, to characterize the emergent cohort of women endorsing premenstrual suicidality, and to evaluate for any differences between groups related to early trauma and adversity. Concurrent SI was reported in 26% of women in our sample who reported premenstrual symptoms. We additionally found several significant differences between the group of women who reported PMSI and the group who did not. In preliminary analyses, the group who endorsed PMSI were younger, more likely to be single and unemployed or underemployed, and less likely to have children. Additionally, the group who endorsed PMSI was more likely to report a history of depression, anxiety, psychiatric hospitalization, and prior suicide attempt as well as elevated scores on current measures of depressive and anxious symptoms and interference from their menstrual symptoms in daily functioning. Finally, women who reported PMSI were more likely to have higher trauma burdens (ACEs > 3) than those who did not. Although the variables above were associated with PMSI in preliminary bivariate analysis, the final logistic regression model indicated that three key variables distinguished women who reported PMSI from those who did not: history of a depression diagnosis, severity of their current depressive symptoms, and a higher burden of childhood traumatic events.

That a quarter of our sample of women who endorsed premenstrual symptoms reported concurrent SI supports the emerging clinical evidence that premenstrual mood symptoms with SI is not a rare occurrence. A recent meta-analysis of data from prior studies with varied methodologies. Affirmed that PMDD and PMS are both associated with increased risk of reporting SI [4]. The process of isolating dynamic risk factors related to increased vulnerability to suicide is critically important to the implementation of suicide prevention strategies. A potentially cyclic and, therefore, predictable period of increased vulnerability in women with co-occurring premenstrual symptoms is of major clinical significance to all providers who treat women with premenstrual symptoms, and it opens up further avenues of clinical inquiry. Specifically, providers encountering women with premenstrual symptoms may consider screening for and monitoring suicidality concomitant with those symptoms, especially among those with a depression and trauma history.

The differences found between groups support the hypothesis that additive risk factors such as unemployment and single status without children contribute to the vulnerability of the group of women reporting concurrent premenstrual symptoms with SI. While it can be argued that these additive risk factors may contribute to a more or less symptomatic group overall, the specific endorsement of new or worsening suicidality only in the premenstrual period cannot easily be explained by this hypothesis. An alternate hypothesis is that the constellation of interrelated risk factors seen in this group have a unifying and confounding original risk factor, which brings us to the discussion of our final major findings.

Depression appeared to play a key role in predicting PMSI. Congruent with our findings, other research has shown that more severe depressive symptoms in the luteal phase and psychiatric co-morbidity, especially a depression diagnosis, are associated with the suicidal risk linked to premenstrual symptoms [24, 25]. In contrast, a recent systematic review found that psychiatric co-morbidities did not account for SI associated with premenstrual symptoms [26]. However, these authors note that a limitation of their review was that women with existing psychiatric disorders had been excluded from most of the studies. In a national study of women’s suicidal risk, severity of depression and history of a depression diagnosis were identified as key predictors of suicidal ideation and suicide attempts for women of all reproductive stages [27]. Although suicidal behavior associated with premenstrual symptoms may be uniquely different than other forms of suicidal behavior, our results suggest that the role of past and current depression cannot be ignored.

Analysis of our study data demonstrates that women with 3 or more adverse childhood adverse events were 3.45 times more likely to report new or worsening SI concurrent with premenstrual symptoms. While our study is exploratory, this finding may support Eisenlohr-Moul et al’s^10^ work demonstrating that women with histories of abuse have increased hormone sensitivity. This finding may also further support a hypothesis that higher burdens of trauma may impact the degree of sensitivity to ovarian steroid hormones and the severity of symptoms provoked by hormonal shifts in vulnerable women. Further study is necessary to explore this hypothesis, as well as to better understand the mechanisms by which early adversity and trauma may contribute to a dysregulated response to estrogen and progesterone and shape the pathophysiology of hormone sensitivity.

The current study is a further demonstration that a subset of women experience severe premenstrual symptoms related to functional impairment and serious psychiatric symptoms such as SI. In our study, although this cohort of women with severe premenstrual symptoms was more commonly encountered in inpatient and outpatient psychiatric settings, they could also be detected in obstetric outpatient settings where they may be encountered in routine obstetric care. Perhaps the most significant limitation of this data is intrinsic to the nature of all cross-sectional data: it is subject to recall bias. Firstly, we cannot state that the reported premenstrual symptoms coincided with hormonal shifts, as we have no confirmatory serum hormone or menstrual cycle tracking data available. Furthermore, women who report PMSI have higher scores on current symptoms scales of depression and anxiety and are more likely to be encountered in the inpatient psychiatric hospital setting; it is plausible that the current severity of their symptoms may be contributing to a negative recall bias. Notably, severity of current depressive symptoms was the most significant contributor to PMSI in our final logistic regression. Additionally, as only 5% of women reporting PMSI in our study were encountered in the outpatient obstetric-gynecology clinic setting while the rest were encountered in psychiatric outpatient or inpatient settings, generalizability to women who are not encountered in such psychiatric settings may be limited. Finally, the presence of premenstrual symptoms and PMSI was captured using language derived from routine clinical assessment rather than administration of validated scales in this self-report cross-sectional study.

Prospective cohort studies consisting of groups defined by validated clinical scales and strict diagnostic criteria are needed to address these limitations and validate the findings of the present study, and there is an urgent need for the study of the premenstrual period given its potential as a dynamic risk factor for suicidal behavior. Additionally, further research is needed to illuminate the role of endocrine hormones and trauma in the developmental pathophysiology of PMS and PMDD in order to define targets for interventions, including psychopharmacological therapeutics. Although further research is necessary, the present study supports the importance of screening for suicidality in patients with premenstrual symptoms encountered across clinical settings.

## Conclusions

A subset of women with premenstrual symptoms may also experience concurrent SI, further highlighting the urgency of diagnosing and adequately treating this often overlooked disorder. Assessment of depression history, current depressive symptoms, and trauma history may be essential aspects of ongoing primary and women’s health care in light of their significant associations to premenstrual suicidal ideation. Our findings support that there is a need for greater clinical awareness of menstrual mood disturbances, particularly amongst women with comorbid diagnoses and histories of trauma.

## Supplementary Information

**Additional file 1.**

## Data Availability

The data generated and analyzed during this study are not publicly available.

## Abbreviations

SI: Suicidal ideation
PMS: Premenstrual syndrome
PMDD: Premenstrual dysphoric disorder
PMSI: Premenstrual suicidal ideation
GAD-7: Generalized Anxiety Disorder 7-item scale
PHQ-9: Patient Health Questionnaire
ACEs: Adverse Childhood Experiences Questionnaire
CI: Confidence interval
SD: Standard deviation
SE: Standard error
OR: Odds ratio
