# Supplementary material for: Clinical correlates of women endorsing premenstrual suicidal ideation: a cross-sectional study
Source: Biopsychosoc Med. 2022 Nov 8;16:23. doi: 10.1186/s13030-022-00252-3 (PMC9644454; doi:10.1186/s13030-022-00252-3)
Supplement: Supplementary file 1 — Additional file 1. [file 13030_2022_252_MOESM1_ESM.docx]

Supplementary Table 1: Demographics of Menstrual Mood questionnaire completers vs. non-completers

| Variables | No menstrual mood data frequency (%) | Completed menstrual mood data frequency (%) | p |  |
| --- | --- | --- | --- | --- |
|  |  |  |  |  |
| **Age, n = 295** |  |  | 0.485 |  |
| 18 to 25 | 9 (18%) | 58 (24%) |  |  |
| 26 to 35 | 16 (31%) | 84 (34%) |  |  |
| 36 to 45 | 12 (24%) | 60 (25%) |  |  |
| 46 to 55 | 5 (10%) | 18 (7%) |  |  |
| 55+ | 9 (18%) | 24 (10%) |  |  |
|  |  |  |  |  |
| **Setting, n = 286** |  |  | 0.056 |  |
| Inpatient Psychiatry | 9 17%) | 73 (31%) |  |  |
| Outpatient Psychiatry | 20 (39%) | 58 (25%) |  |  |
| Outpatient Ob-gyn | 23 (44%) | 103 (44%) |  |  |
|  |  |  |  |  |
| **Ethnicity, n = 275** |  |  | 0.653 |  |
| Not Hispanic or Latino | 36 (75%) | 163 (72%) |  |  |
| Hispanic or Latino | 12 (25%) | 64 (28%) |  |  |
|  |  |  |  |  |
| **Race, n = 249** | | | 0.505 |  |
| Caucasian | 23 (51%) | 118 58%) |  |  |
| African-American | 13 (29%) | 41 (20%) |  |  |
| American Indian | 0 (0%) | 4 (2%) |  |  |
| Asian Indian | 2 (4%) | 18 (9%) |  |  |
| East or South Asian | 7 (16%) | 22 (11%) |  |  |
| Pacific Islander | 0 (0%) | 1 (1%) |  |  |
| Other |  |  |  |  |
|  |  |  |  |  |
| **Marital Status, n = 300** |  |  | 0.250 |  |
| Single | 16 (29%) | 91 37%) |  |  |
| Married | 30 (55%) | 93 (38%) |  |  |
| Committed relationship | 3 (6%) | 33 (14%) |  |  |
| Separated | 1 (2%) | 6 (2%) |  |  |
| Widowed | 2 (4%) | 6 (2%) |  |  |
| Divorced | 3 (6%) | 16 (7%) |  |  |
|  |  |  |  |  |
| **Has biological children, n = 299** | |  | 0.395 |  |
| Yes | 31 (57%) | 125 (51%) |  |  |
| No | 23 (43%) | 120 (51%) |  |  |
|  |  |  |  |  |
| **Education, n = 287** |  |  | 0.286 |  |
| Less than High School | 2 (5%) | 1 (<1%) |  |  |
| High School | 12 (27%) | 65 (27%) |  |  |
| GED | 0 (0%) | 7 (3%) |  |  |
| 2 year college | 8 (18%) | 53 (22%) |  |  |
| 4 year college | 10 (23%) | 64 (26%) |  |  |
| Master's | 9 (21%) | 40 (17%) |  |  |
| Professional (MD, JD) | 3 (7%) | 12 (5%) |  |  |
| PhD | 0 (0%) | 1 (<1%) |  |  |
|  |  |  |  |  |
| **Income, n = 272** |  |  | 0.163 |  |
| < $15,000 - 20,999 | 6 (14%) | 51 (22%) |  |  |
| $21,000 - $50,999 | 6 (14%) | 29 (13%) |  |  |
| $51,000 - $100,999 | 15 (36%) | 48 (21%) |  |  |
| $101, 000 - >$150,000 | 15 (36%) | 102 (44%) |  |  |
|  |  |  |  |  |
| **Employment, n = 279** |  |  | 0.603 |  |
| Homemaker | 7 (17%) | 26 (11%) |  |  |
| Unemployed | 10 (24%) | 62 (26%) |  |  |
| Employed Occasionally | 0 (0%) | 9 (4%) |  |  |
| Employed Part-time | 8 (19%) | 49 (21%) |  |  |
| Employed Full-time | 17 (41%) | 91 (38%) |  |  |
|  |  |  |  |  |
| **Lifetime history of depression, n = 283** | |  | 0.791 |  |
| Yes | 20 (50%) | 127 (52%) |  |  |
| No | 20 (50%) | 116 (48%) |  |  |
|  |  |  |  |  |
| **Lifetime history of anxiety, n = 272** | |  | 0.926 |  |
| Yes | 14 (41%) | 96 (40%) |  |  |
| No | 20 (59%) | 142 (60%) |  |  |
|  |  |  |  |  |
| **History of suicide attempt, n = 275** | |  | 0.307 |  |
| Yes | 6 (18%) | 64 26%) |  |  |
| No | 27 (82%) | 178 (74%) |  |  |
|  |  |  |  |  |
| **History of psychiatric hospitalization, n = 275** | |  | 0.065 |  |
| Yes | 8 (24%) | 99 (41%) |  |  |
| No | 25 (76%) | 143 (59%) |  |  |
|  |  |  |  |  |
| **Menstrual status, n = 280** | | | 0.336 |  |
| Menstruating | 21 (50%) | 138 (58%) |  |  |
| Not menstruating | 21 (50%) | 100 (42%) |  |  |
|  |  |  |  |  |
| **Menstrual regularity, n = 160** | | | 0.352 |  |
| Regular periods | 17 (81%) | 99 (71%) |  |  |
| Irregular periods | 4 (19%) | 40 (29%) |  |  |
